# Origin of age softening in the refractory high-entropy alloys

**DOI:** 10.1126/sciadv.adj1511

**Published:** 2023-12-08

**Authors:** Junliang Liu, Bo-Shiuan Li, Hazel Gardner, Yilun Gong, Fengxian Liu, Guanze He, Michael Moorehead, Calvin Parkin, Adrien Couet, Angus J. Wilkinson, David E. J. Armstrong

**Affiliations:** ^1^ Department of Materials, University of Oxford, Parks Road, Oxford OX1 3PH, UK.; ^2^ Department of Nuclear Engineering and Engineering Physics, University of Wisconsin–Madison, 1500 Engineering Drive, Madison, WI 53706, USA.; ^3^ Department of Mechanical and Electromechanical Engineering, National Sun-Yat Sen University, No. 70 Lien-hai Rd., Kaohsiung, Taiwan.; ^4^ UKAEA, Culham Science Centre, Abingdon, Oxfordshire, UK.; ^5^ Computational Materials Design Department, Max-Planck Institut für Eisenforschung GmbH, Max-Planck-Straße 1, 40237 Düsseldorf, Germany.; ^6^ Department of Mechanics of Solids, Surfaces and Systems, Faculty of Engineering Technology, University of Twente, Drienerlolaan 5, 7522NB Enschede, Netherlands.; ^7^ Idaho National Laboratory, Idaho Falls, ID, 83415, USA.

## Abstract

Refractory high-entropy alloys (RHEAs) are emerging materials with potential for use under extreme conditions. As a newly developed material system, a comprehensive understanding of their long-term stability under potential service temperatures remains to be established. This study examined a titanium-vanadium-niobium-tantalum alloy, a promising RHEA known for its superior high-temperature strength and room-temperature ductility. Using a combination of advanced analytical microscopies, Calculation of Phase Diagrams (CALPHAD) software, and nanoindentation, we investigated the evolution of its microstructure and mechanical properties upon aging at 700°C. Trace interstitials such as oxygen and nitrogen, initially contributing to solid solution strengthening, promote phase segregation during thermal aging. As a result of the depletion of solute interstitials within the metal matrix, a progressive softening is observed in the alloy as a function of aging time. This study, therefore, underscores the need for a better control of impurities in future development and application of RHEAs.

## Teaser

Impurity elements were observed to induce softening in a newly designed TiVNbTa engineering alloy at high temperatures.

## INTRODUCTION

High-entropy alloys (HEAs) represent a new class of alloys that consist of more than four alloying elements in close to equiatomic concentrations (*1*). The diverse compositional possibilities of HEAs, combined with appropriate heat treatment conditions, offer the potential to develop new alloys with a combination of remarkable properties such as outstanding mechanical properties, high-hydrogen absorption capacity, and robust resistance to corrosion and radiation (*2*–*5*). The refractory HEAs (RHEAs) based on refractory elements were proposed by Senkov *et al.* (*6*) as a new class of high-temperature structural alloys that might surpass the capabilities of nickel-based superalloys. RHEAs are emerging as prominent contenders in diverse industries, notably in the nuclear and aerospace sectors (*5*, *7*).

The presence of intermetallic compounds, such as brittle Laves phases, typically reduces the ductility of RHEAs (*8*), prompting extensive efforts to develop single-phase RHEAs for high-temperature applications (*5*, *7*, *8*, *9*, *10*, *11*, *12*). The equiatomic Ti-V-Nb-Ta alloy is one of the most attractive RHEAs, which solidifies as a single-phase body-centered cubic (BCC) microstructure with heterogeneous elemental distribution and exhibits excellent high-temperature strength and room-temperature ductility (*13*–*17*). Equimolar Ti-V-Nb-Ta has an extremely high compressive yield strength, 1273 MPa at room temperature and 688 MPa at 900°C (*14*). By comparison, conventional high-temperature Ni-base superalloys notably soften above 600°C, with the compressive yield strength dropping to ~200 MPa at 900°C (*18*). Equimolar Ti-V-Nb-Ta also exhibits impressive tensile ductility, ~14% at room temperature (*16*), and a relatively low brittle-to-ductile transition temperature between −47° and −27°C, comparable to BCC metals and stainless steels for engineering applications (*15*, *19*).

Despite these advances, the bulk of experimental work studying the Ti-V-Nb-Ta system and other RHEAs has focused on materials under the as-cast or homogenized conditions. These conditions may not accurately represent their long-term structural/mechanical stability at service temperatures. As a newly developed material system, a comprehensive understanding of their long-term stability under potential service temperatures remains to be established. Some RHEAs showing single-phase structure under the as-cast condition begin to phase separate and form precipitates when heat treated at intermediate temperatures (700° to 800°C) (*20*), which are close to the operating temperatures of some potential applications for RHEAs, such as in core components of the next-generation nuclear reactors (*5*, *21*, *22*). In addition, some recent studies have highlighted the substantial impact of oxygen and nitrogen additions, in the range of 1 to 3 atomic % (at %) (*3*, *17*, *23*), on the mechanical properties of HEAs. For instance, Cui *et al.* (*3*) demonstrated that a Ti-V-Hf-Nb alloy with ~2 at % oxygen additions can reach a yield strength of 1.5 GPa, while maintaining substantial ductility. Similarly, Lei *et al.* (*23*) reported that by introducing 2 at % N, the tensile strength of a Ti-Zr-Hf-Nb alloy can surge by over 70%. These interstitials are often incorporated at trace levels during processing RHEAs and can accumulate further in service. However, a notable knowledge gap remains, especially regarding the behavior of these oxygen/nitrogen interstitials during high-temperature aging. The following questions remain unanswered: Will these interstitials continue to contribute positively to the mechanical properties during the long-term service? And will they affect the phase stability or aid in phase segregation at in-service temperatures? These ambiguities necessitate further investigation.

In the present investigation, Ti-V-Nb-Ta samples were subjected to thermal aging for various durations at 700°C. The microstructure and mechanical properties of Ti-V-Nb-Ta before and after thermal aging were revealed by a combination of state-of-the-art microstructural characterization and nanoindentation techniques. Results indicate that impurity interstitials at the trace levels within the solid solution encourage the formation of α-Ti precipitates in the Ti-V-Nb-Ta alloy when aged at high temperature, which undermines the effects of interstitial solid solution strengthening and leads to softening.

## RESULTS

### Nanoindentation hardness and modulus

**Fig. 1.**
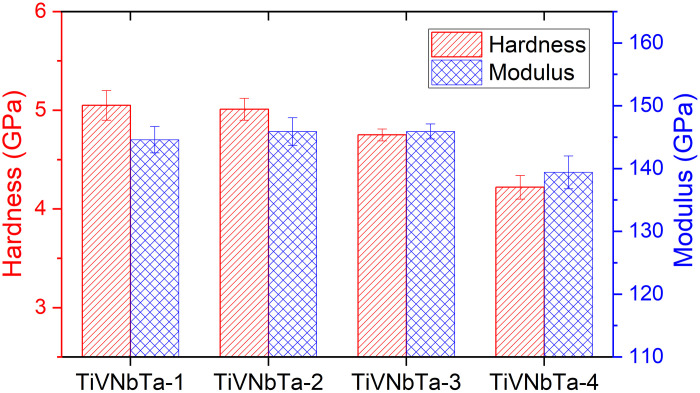
Nanoindentation hardness. Samples after different heat treatments are described in Table 1.

Sample codes and nanoindentation hardness and modulus results for specimens subjected to different heat treatments are summarized in Table 1 and Fig. 1, respectively. The homogenized Ti-V-Nb-Ta exhibits a hardness of 5.05 GPa, which is comparable to some conventional structural materials, such as 301SS (*24*). After homogenization, the Ti-V-Nb-Ta samples displayed a characteristic age softening behavior, with the material progressively softening over the thermal aging period (Fig. 1). The hardness of the 40-day aged sample, TiVNbTa-4, decreased by approximately 16% relative to the as-homogenized state, dropping to 4.22 GPa, as shown in Fig. 1.

**Table 1.** Samples of different heat treatments for micromechanical and microstructural analysis.

Sample ID	TiVNbTa-1	TiVNbTa-2	TiVNbTa-3	TiVNbTa-4
Conditions	Homogenized at 1200°C for 7 days	Homogenized and then aged at 700°C for 1 day	Homogenized and then aged at 700°C for 5 days	Homogenized and then aged at 700°C for 40 days

### Microstructure

**Fig. 2.**
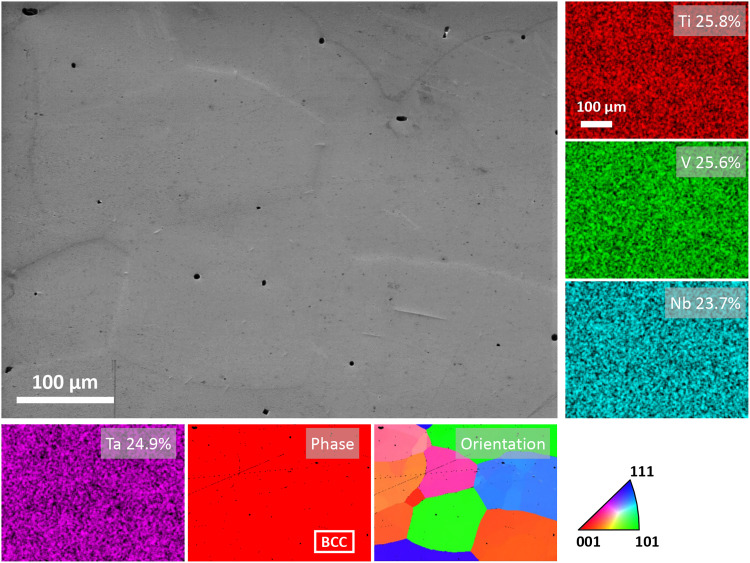
Colocated SEM, EDX, and EBSD maps for TiVNbTa-1. Elemental concentration is directly reflected by the color intensity in the EDX maps and the unit for element concentrations labeled in the EDX maps is atomic %.

After homogenization at 1200°C for 7 days, the Ti-V-Nb-Ta sample exhibits a single-phase BCC structure with a fully homogeneous elemental distribution and a grain size greater than 150 μm, Fig. 2. Observation of similar homogenized microstructure was also reported by Lee *et al.* (*14*) on a similar Ti-V-Nb-Ta alloy and Liu *et al.* (*25*) on a Ti-V-Cr5-Nb-Ta alloy. The black dots in the scanning electron microscopy (SEM) image and the electron backscatter diffraction (EBSD) maps (Fig. 2) likely originate from the pores in the alloy rather than any secondary phases because no obvious aggregation of alloying elements can be seen from the energy-dispersive x-ray (EDX) maps. The phase identified in the homogenized sample concurs with the calculated phase diagram (*13*), which predicts an equilibrium BCC structure in Ti-V-Nb-Ta at 1200°C. When aged at 700°C, the grain morphology and alloying elements distribution were not observed to exhibit obvious changes at the submicrometer scale even after 40 days (fig. S1).

**Fig. 3.**
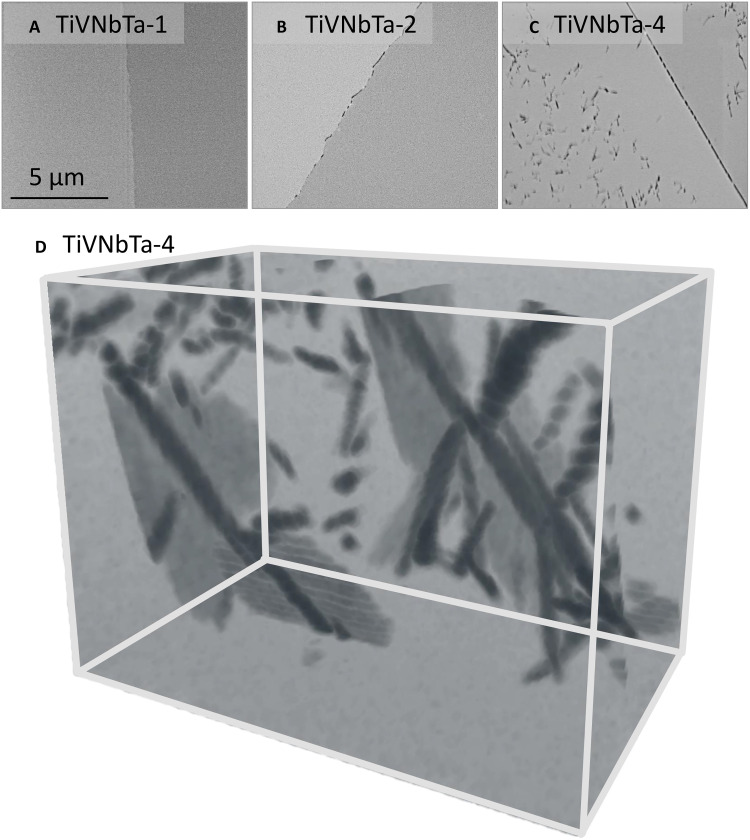
HAADF-STEM and 3D FIB-SEM characterization. (**A** to **C**) HAADF-STEM images showing the precipitation in samples of different heat treatments. (**D**) 3D FIB/SEM visualization of the morphology of intragranular precipitates in the 40-day aged sample. The dimension of the visualized volume is ~1.67 μm by 1.12 μm by 1.05 μm.

The microstructure evolution at the nanoscale as a function of aging time in Ti-V-Nb-Ta alloy is displayed in Fig. 3 (A to C). The high-angle annular dark-field scanning transmission electron microscopy (HAADF-STEM) images in Fig. 3 (A to C) show *Z*-contrast in which the precipitates with lower average atomic mass relative to the surrounding matrix will exhibit darker contrast. Under the homogenized condition (TiVNbTa-1), samples were observed to be free of secondary phase precipitates. Under the 1-day aging condition (TiVNbTa-2), a few precipitates are seen to nucleate at grain boundaries (Fig. 3B). Upon further aging, in addition to grain boundary precipitates, precipitation also occurs within the grains (Fig. 3C). Another noticeable feature of the 40-day aging sample is the “denuded zones” or “precipitate-free zones (PFZs)” present around 3 μm adjacent to the grain boundary (Fig. 3C). Three-dimensional focused ion beam/SEM (3D FIB/SEM) sequential sectioning was used to analyze the morphology of these intragranular precipitates (Fig. 3D). It is seen that these intragranular precipitates were formed with a volume fraction of approximately 3%. By looking at the 3D image, three distinct morphologies of precipitates are observed in the 40-day aging sample, as shown in Fig. 3D: plate-like, rod-like, and particle-like. The particle- and rod-like precipitates show dark contrast, while the plate-like precipitates show lighter contrast. These intragranular precipitates are clearly aligned to specific directions within each grain that becomes more obvious during the thermal aging. These microstructure features have been widely reported in metastable β-Ti alloys (*26*, *27*).

**Fig. 4.**
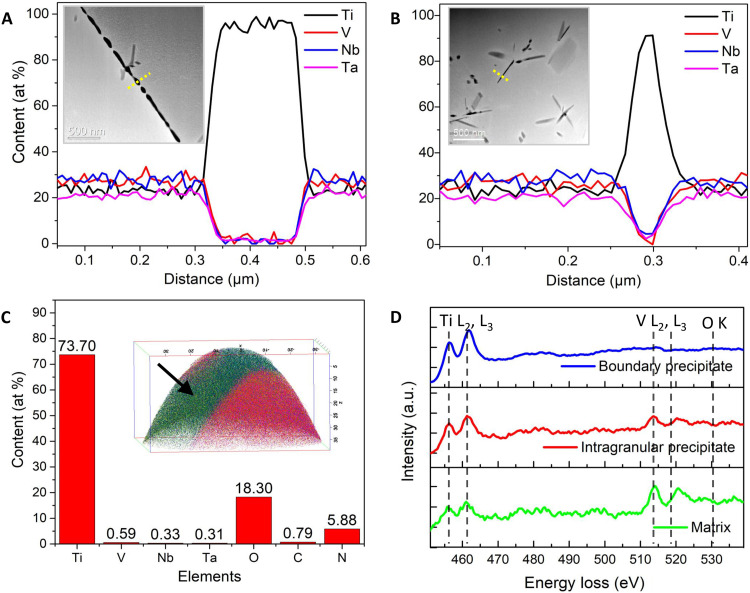
Composition analysis of precipitates in the sample TiVNbTa-4. (**A** and **B**) STEM-EDX line scans and (**C**) APT atomic-scale analysis. The yellow dashed lines in the inserted STEM images in (A) and (B) indicate the locations for EDX line scans. The arrow in (C) shows the precipitate in the 3D APT dataset. (**D**) EELS Ti-L_2,3_ and O-K core-loss spectra for precipitates and the matrix after subtraction of background. a.u., arbitrary units.

The chemical composition of these precipitates was determined using a combination of analytical techniques, shown in Fig. 4. The EDX line scans from the 40-day aged sample indicate the intergranular and intragranular precipitates are both Ti-enriched, with >90 at % Ti, although their morphology and size are obviously different. Peak overlapping often occurs in the analysis of EDX characteristic peaks for light elements (*Z* < 10), leading to inaccurate identification and quantification of these corresponding elements, thus only heavy elements (Ti, V, Nb, and Ta) were included in the EDX quantification analysis. Atom probe tomography (APT) technique offers much more sensitivity compared to EDX for the detection of light elements, such as nitrogen and oxygen. For this, APT was further used to analyze the chemical composition of these precipitates at the atomic scale. Figure 4C shows an example of an APT tip lifted from the 40-day aged sample (TiVNbTa-4) covering part of a precipitate and the surrounding matrix. The APT results indicate that the precipitates are enriched with titanium, oxygen, and nitrogen, which accounts for 73.7, 18.3, and 5.88 at %, respectively, of the precipitate composition. APT was also used to analyze the content of major interstitials in the matrix, e.g., 1.12 to 1.3 at % (C, N, and O) in the 1-day aged sample (TiVNbTa-2) decreases to 0.56 to 0.64 at % in the 40-day aged sample (TiVNbTa-4) (table S1).

The electron energy loss spectroscopy (EELS) technique can provide information about local chemical compositions, valence states, or crystallographic structure. The energy shift of the Ti-L_2,3_ edge and its specific fine structure have been used as fingerprint to distinguish the chemical nature of titanium in its nitrides and oxides (*28*, *29*). The Ti-L_2,3_ edges in Ti nitrides are observed to shift to higher energies relative to their base Ti alloy (*28*). Stoyanov *et al.* (*29*) reported that the Ti-L_2,3_ white lines split into four peaks in Ti oxides with O > 50 at %. To confirm the chemical nature of the precipitates, either a Ti(O, N)*_x_* compound or a metallic precipitate, we acquired EELS spectra from the 40-day aged sample (TiVNbTa-4) and plotted in Fig. 4D. The Ti-L_2,3_ white lines of the precipitates are observed with similar shape to that of the matrix. This means that the solubility of oxygen or nitrogen in the quantities seen here (18 at % for oxygen and 6 at % for nitrogen from APT analysis) does not strongly affect the chemical nature of Ti in the precipitates; that is, the precipitates formed in the 40-day aged sample are metallic in nature rather than compounds. The observed minor edge shift in the Ti-L_2,3_ edges for boundary precipitates, in comparison to intragranular precipitates and the matrix, could potentially be ascribed to subtle differences in the lattice structure (*30*). It is also worth noting that the core-loss spectra from the intragranular precipitate show V-L_2,3_ white lines, which probably arise from the matrix covering that precipitate.

**Fig. 5.**
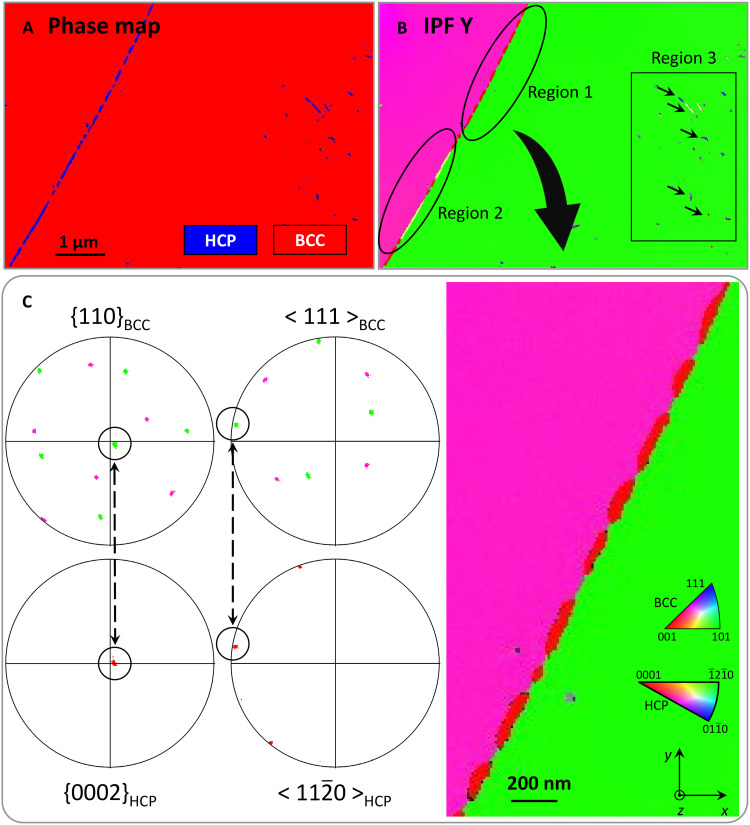
TKD analysis of boundary precipitates. (**A** and **B**) TKD phase and orientation (IPF Y) maps show the microstructure of the sample TiVNbTa-4. Regions 1 to 3 labeled in (B) are used for grain-to-grain orientation relationship analysis. (**C**) TKD orientation relationship analysis in the region 1. The color of points scattered to different angles in the pole figures in (C) is displayed in the same color scheme in (B). The arrows in (C) indicated the aligned planes and directions for the two phases.

Transmission Kikuchi diffraction (TKD) orientation mapping was used to identify the crystal structure of these precipitates at the nanoscale. Both the grain boundary precipitates and intragranular precipitates were clearly resolved (Fig. 5). The matrix was indexed as a BCC structure, and the precipitates were indexed as a hexagonal close-packed (HCP) structure. Considering the previous APT and EELS chemical analysis, these precipitates are determined to be α-Ti phase with solid solution oxygen and nitrogen.

The TKD inverse pole figure (IPF) map is colored on the basis of the crystal orientation along the selected sample direction. As shown in the IPF maps in Fig. 5B and fig. S2, the boundary precipitates mainly exhibit two orientations, while the intragranular precipitates exhibit six different orientations. These precipitates of different orientations were sorted into three regions (Fig. 5B), and TKD stereographic analysis was performed in each region to identify the orientation relationship(s) between the precipitates and the matrix. It is worth addressing that the scatter points in the pole figures are colored using the same color scheme for the IPF Y maps; thus, the green points in the pole figures are scattered from the grain on the right-hand side, the purple points from the left-hand side grain, and the red ones from the boundary precipitates. The pole figures and the corresponding IPF Y map for region 1 are shown in Fig. 5C. For this mapped area, the (0002)_HCP_ and one of the 6 {110}_BCC_ planes of the right-hand matrix grain are coincident, and [11$\bar{2}$0]_HCP_ directions is parallel to one of the 〈111〉_BCC_ directions, concurring with the Burgers orientation relationship (BOR), (0002)_HCP_ ǁ {110}_BCC_ and [11$\bar{2}$0]_HCP_ ǁ <111>_BCC_, for BCC to HCP transformations (*31*). All the boundary precipitates in region 1 grew out of the same parent BCC grain on the right-hand side of the boundary and exhibit the same orientation with the (0002)_HCP_ normal to the *y* direction. TKD stereographic analysis for region 2 shows that the boundary precipitates in region 2 were transformed from the left-hand side BCC grain and exhibit the same BOR to region 1 (fig. S3).

In BOR, each (0002)_HCP_ ǁ {110}_BCC_ plane contains two <111>_BCC_ directions and three <11$\bar{2}$0>_HCP_ directions. One of the <11$\bar{2}$0>_HCP_ directions aligns perfectly with one of the <111>_BCC_ directions, and the second <11$\bar{2}$0>_HCP_ direction is ∼11° (depending on the relative lattice parameters of the BCC and HCP phases) away from the second <111>_BCC_ directions. The final <11$\bar{2}$0>_HCP_ axis shows no close alignment with a 〈111〉_BCC_ direction. This gives the 12 possible BOR variants. These results indicate that the formation of boundary precipitates obeys the BOR. Here, the boundary precipitates that grew out of the same parent BCC grain exhibit the same orientation, indicating that 1 of the 12 orientation variants was dominating in the growth of boundary precipitates.

**Fig. 6.**
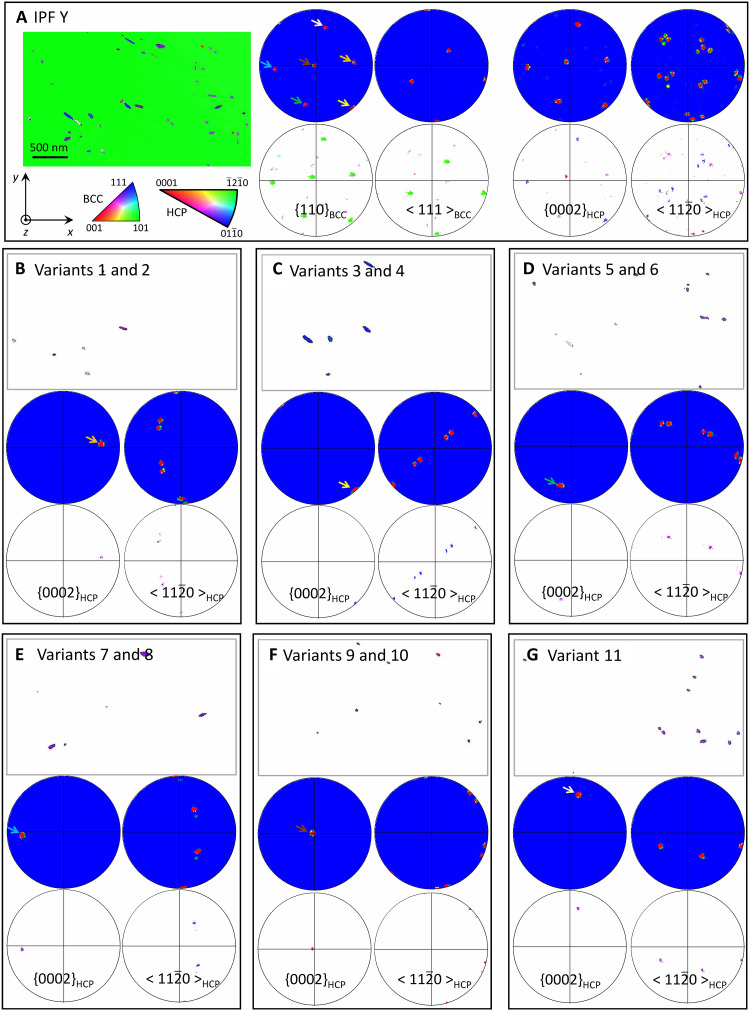
TKD analysis of intragranular precipitates. (**A**) IPF Y map showing all the precipitates within the mapped region and the corresponding scattered and contoured pole figures. (**B** to **G**) IPF Y maps showing precipitates of different BOR variants and their corresponding pole figures. The arrows indicate the aligned {0002}_HCP_ and {110}_BCC_ poles.

The orientation analysis for the intragranular precipitates is illustrated in Fig. 6. To better show the alignment, we exhibit both the scattered and contoured poles figures. This mapped area covers a single grain with the (110)_BCC_ normal to the *y* direction. As shown in Fig. 6A, the six {110}_BCC_ planes give rise to precipitates of six (0002)_HCP_ orientations, and 11 BOR variants were identified from the grain-by-grain stereographic analysis (Fig. 6, B to G). The variants are divided into six subgroups based on the alignment between (0002)_HCP_ and {110}_BCC_ planes (Fig. 6, B to G). For the two specific BOR variants within the same subgroup [or showing the same (0002)_HCP_ ǁ {110}_BCC_ plane], a mismatch ~11° between the <11$\bar{2}$0>_HCP_ poles can be expected, and this can be seen in the corresponding pole figures (Fig. 6, B to G). It is worth noting that the TKD pixel size/spatial resolution plays an important role in identifying the precipitates of different variants. Only six BOR variants can be identified in maps in which a 10-nm TKD pixel size was used, while 11 variants can be seen when the pixel size was 5 nm. The precipitate of the 12th variant is probably too small to be detected in the thin foil. The analysis results suggest that there is no preferred selection of BOR variants in the formation of intragranular precipitates and, globally, all the 12 variants can nucleate.

### Calculated equilibrium phase diagram

**Fig. 7.**
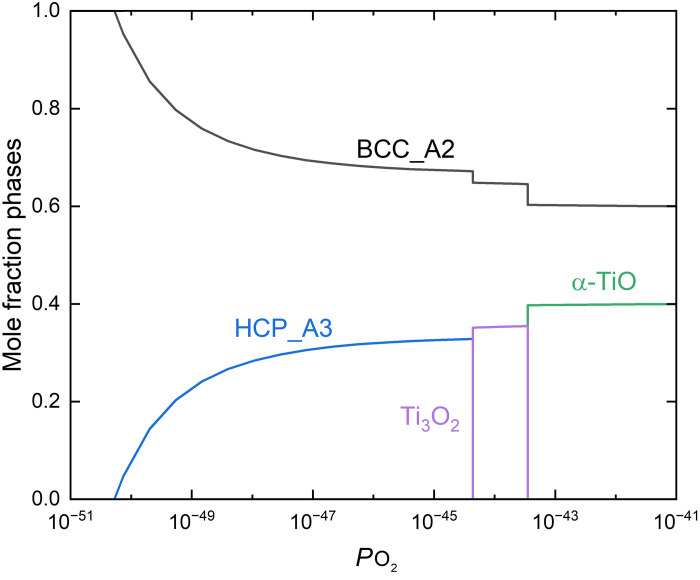
Calculated equilibrium phase diagram. The equilibrium phase fraction in the Ti-V-Nb-Ta system at 700°C as a function of oxygen partial pressure (*P*o_2_).

Yao *et al.* (*13*) and Lee *et al.* (*14*) have used the Calculation of Phase Diagrams (CALPHAD) method to calculate the equilibrium phase diagram for the Ti-V-Nb-Ta system and predicted a single-phase BCC microstructure at 700°C. However, the presence of oxygen (which is a common contaminant due to its high solubility in and high affinity for titanium and vanadium) has not been taken into account in the calculations by previous researchers (*13*, *14*). Figure 7 shows the equilibrium molar fractions of phases calculated as a function of oxygen partial pressure in the Ti-V-Nb-Ta system at 700°C. Calculations were performed using Thermo-Calc (v.2023b) coupled with the Ti alloy database TCTI2 (*32*). As shown in Fig. 7, small additions of oxygen can facilitate the formation of the HCP-Ti phase, which concurs with our experimental observations, i.e., oxygen is an α-stabilizer. With further increasing oxygen content, the fraction of the BCC phase continues decreasing in favor of the formation of different types of oxides. A plot showing the equilibrium phase fractions as function of oxygen content in mole fraction in the Ti-V-Nb-Ta system at 700°C is shown in fig. S4.

## DISCUSSION

### Mechanisms underpinning age softening in RHEAs

The CALPHAD calculations indicate that oxygen can substantially affect the phase stability in Ti-V-Nb-Ta. In the absence of oxygen, equimolar Ti-V-Nb-Ta is predicted to exhibit a single-phase BCC structure, while trace levels of oxygen facilitate the formation of the α-Ti phase at the aging temperature, consistent with oxygen’s role as an α-stabilizer in Ti alloy (*33*). As nitrogen is also an α-stabilizer in Ti alloys, it may play a similar role to oxygen in facilitating the formation of α-Ti phase in the Ti-V-Nb-Ta. Grain boundary α-Ti precipitates are observed to nucleate initially, followed by the formation of intragranular precipitates and the emergence of PFZs adjacent to the grain boundaries. The experimental characterization and equilibrium phase calculations lead to the inference that the formation of PFZ in the Ti-V-Nb-Ta system is the result of diffusion of Ti and major interstitials (O and N) to grain boundaries, with subsequent formation of nitrogen- and oxygen-rich α-Ti precipitates at the aging temperature. These boundary precipitates drain the adjacent matrix from solute oxygen and nitrogen, and, hence, an oxygen/nitrogen-depleted PFZ arises, with coarse particles at the boundaries. The precipitation, at the boundaries or intragranular, all undergoes the Burgers relationship with (0002)_HCP_ ǁ {110}_BCC_ and [11$\bar{2}$0]_HCP_ ǁ <111>_BCC_. The difference between these two types of precipitation is the selection of orientation relationship variants. The intragranular precipitates exhibit a finely interleaved lamellae structure that is due to the occurrence of 12 possible crystallographic variants of α-Ti in a single-parent grain. Since grain boundary precipitates can form with the Burgers relationship with one of the grains on either side of the boundary, a choice of 24 variants is available, of which up to two variants can dominate in a given grain boundary.

While it is true that secondary phase precipitates have been found to enhance hardness in some Ti alloys, the extent of strengthening is largely dependent on both the morphology and volume fraction of the precipitates (*34*, *35*) and the precipitate/matrix interface structure (*36*). We theoretically calculated the hardening effects resulting from the precipitates that are observed in the TiVNbTa-4 sample. The results demonstrate that precipitates with a volume fraction of 3% can contribute to a nanoindentation hardness increase of 217.48 MPa, representing only 5.15% of the overall indentation hardness of the TiVNbTa-4 sample. It is also shown that rod-like precipitates can make dominant contributions to the precipitation strengthening relative to the plate- and particle-like precipitates. Our calculations align with the observations made by Dehghan-Manshadi and Dippennar (*35*). Interpreting from their data, the formation of approximately 7% α-phase precipitates in Ti alloys results in a microhardness increase of around 20 Hv that corresponds to less than 6.5% of the total microhardness. Consequently, the contributions to hardness from these precipitates observed in RHEA in the current are expected to be minimal and could easily be overshadowed by reductions because of other mechanisms, such as decreased solid solution strengthening.

There have been extensive efforts to investigate the strengthening mechanisms in RHEAs (*17*, *37*, *38*), and the dominant strengthening mechanism in single-phase RHEAs was mostly reported to be the solid solution hardening (*14*). However, unlike conventional alloys, quantifying the effect of solid solution strengthening on HEAs is challenging because of the inability to distinguish between solvent and solute atoms. There are two sources of solid solution hardening in single-phase RHEAs: (i) The random mixing of major alloying elements in the crystal lattice gives rise to local fluctuations and lattice distortions, which introduce energy barriers against dislocation motions and serve as a substitutional solid solution–like strengthening mechanism (*39*); and (ii) Interstitial impurities, such as oxygen and nitrogen, also create high degrees of lattice distortions (*40*) and chemical fluctuations, thereby serving as an interstitial solid solution-like strengthening mechanism. The atomic size difference parameter (δ) has been widely used to assess lattice distortions in HEA (*41*)

$$\delta =100\%\sqrt{\sum_{i=1}^{n}c_{i}{\left(1-\frac{r_{i}}{\sum_{j=1}^{n}c_{j}r_{j}}\right)}^{2}}$$

where *c_i_* represents the atomic fraction of element *i*, *r_i_* is the atomic radius of element *i*, and *n* represents the total number of constituent elements. Chen *et al.* (*42*) reported a linear correlation between the δ value and microhardness for a set of RHEAs. Using atomic radius values from (*43*), we calculated the δ parameters for Ti-V-Nb-Ta alloys with varying oxygen content (table S2). The findings suggest that when adjusting the oxygen content from 1.2 at % (akin to the total content of C, N, and O in the 1-day aged sample) to 0.6 at % oxygen (similar to the total level of C, N, and O in the 40-day aged sample), the level of lattice distortions (or the δ parameter) decreases by approximately 17.6%. This is closely aligned with the observed decrease in hardness between the 1-day and 40-day aged samples (around 16%). This, on the other hand, indicates that the small amount of interstitials can substantially affect the lattice distortions and the observed hardness. Similarly, Lee *et al.* (*17*) reported that additions of 1.6 at % O and N interstitials in the homogenized Ti-V-Nb-Ta system increase the yield strength by about 300 MPa without the observation of precipitates at the nanoscale. Impurity elements such as O and N in the homogenized sample initially occupy the interstitial sites and substantially strengthen the homogenized sample. Upon aging the alloys at temperatures at which the atoms of the precipitate-forming species are sufficiently mobile, the interstitials will partition to the precipitates, leaving the matrix with reduced content of interstitials impurities. Therefore, the strengthening effect from the interstitials decreases with increasing aging time, until the matrix is relatively free of interstitials.

Although the 3% precipitates are observed with limited contributions to hardening in the current study, increasing the volume fraction of these precipitates, particularly the rod-like ones, might offer potent strengthening in HEAs. Exploring ways to strike a dynamic balance between the hardening effect induced by aging precipitates and the softening from reduced solid solution strengthening presents a promising avenue for developing advanced structural materials with stable mechanical properties over long service lifetimes.

### Relevance of this study for processing and applications of RHEAs

Microstructural stability is one of the most important factors for alloys to be used at elevated temperatures. A stable alloy can maintain its properties without substantial changes, even after extended periods of service (*44*). The minor presence of impurity interstitials, e.g., nitrogen and oxygen, in the equimolar Ti-V-Nb-Ta alloy studied was found to introduce uncertainties in its mechanical properties at the aging temperature by facilitating phase segregation at the nanoscale and reducing the solid strengthening effect. Furthermore, the depletion of impurity interstitials in regions adjacent to grain boundaries leads to the formation of PFZs. This spatially confined compositional variation nearby grain boundaries creates very high local electrochemical and mechanical contrast, often affecting the alloy’s corrosion and mechanical properties (*45*–*47*). To improve the stability of RHEAs for high-temperature applications, the impurities must be better controlled. Currently, the predominant production method for RHEAs is arc melting, where contamination present in the melting atmosphere, impurities in the base metals, and adsorbed gases or moisture in the furnace system, particularly oxygen and nitrogen, can be picked up. In the future, suitable operations need to be applied to keep this contamination to a minimum if these materials are to be taken to industrial applications. Scale-up to larger volumes will inevitably mean moving away from arc melting, but the need to control oxygen and nitrogen levels will clearly remain important.

One of the potential applications of RHEAs is for structural components in the next-generation nuclear reactors, including use in fusion reactors, due to their good combination of high-temperature strength and superior radiation resistance (*5*). There have been extensive studies to evaluate the radiation response of RHEAs, e.g., radiation hardening (*5*, *11*, *48*); however, the effects of possible long-term aging softening and the associated precipitate nucleation and growth have been rarely considered. Consider the notable influence of small amounts of impurities on the mechanical properties and phase stability; it is thus suggested that the effects of trace impurities must be taken into consideration in the future studies.

## MATERIALS AND METHODS

### Materials

High-purity (>99.95%) raw elements in slug and foil form were obtained from Alfa Aesar. Ti-V-Nb-Ta RHEA samples were fabricated by vacuum induction melting and then rapidly cooled down in an inert environment to retain the high-temperature structure. After solidification, the ingots were homogenized for 7 days at 1200°C, followed by rapid cooling in an inert environment, to eliminate any microchemical segregation generated during solidification from the liquid state. The homogenized material was then sectioned into squares with a thickness of 3 to 5 mm and a side length of approximately 5 mm, which were then encapsulated in a quartz tube under vacuum for thermal aging heat treatment. The chemistry of the final ingots was characterized using different techniques, inert gas fusion (IGF), combustion infrared detection (CID), and direct current plasma emission spectroscopy (DCPES) techniques as well as inductively coupled plasma optical emission spectrometry (ICP-OES). The final alloy compositions were comparable to the targeted compositions (Table 2). The chemical composition determined using different techniques, such as IGF, CID, DCPES, ICP-OES, and APT, exhibits slight differences. These discrepancies might arise from their differing sample preparation method, sensitivities, and calibrations.

**Table 2.** Measured compositions of homogenized TiVNbTa ingots (*21*). Concentrations are in atomic %.

	Nb	Ta	Ti	V	C	N	O	Si	P	S	Cu
IGF, CID, DCPES	25.6	23	24.6	26.2	0.181	<0.0032	0.091	0.136	0.015	<0.0014	0.116
ICP-OES	25.9	22.4	24.6	26.7	0.098	0.013	0.039	0.03	<0.015	0.006	0.114

To evaluate the long-term thermal stability of the equimolar Ti-V-Nb-Ta alloy, homogenized Ti-V-Nb-Ta samples were aged for 1, 5, and 40 days at 700°C, which is close to the anticipated operating temperatures in several advanced nuclear fission and fusion reactor concepts (*22*). Selected samples for microstructural analysis were ground with abrasive papers from 400 grit down to 2500 grit, followed by diamond paste polishing from 3- to 1-μm grade. Final polishing was performed using colloidal silica with an average particle size of 90 nm for approximately 1 hour. After polishing, the samples were ultrasonically cleaned in methanol for 15 min to remove residual colloidal silica and surface contamination. Microstructures and hardness of selected samples were then characterized by correlative microscopies (SEM, EDX, EBSD, TKD, STEM, and APT) and nanoindentation.

### Nanoindentation tests

Nanoindentation was carried out on an Agilent G200 nanoindenter with a Berkovich diamond indenter tip. Values of hardness and elastic modulus were measured using the continuous stiffness measurement (CSM) method (*49*), with an indentation strain rate at 0.05 s^−1^, a CSM frequency of 45 Hz, a harmonic amplitude of 2 nm, and the Poisson’s ratio of 0.3. An array of 16 indentations with a spacing of 40 μm was performed on each sample. Each indent covers an equilateral triangle area of approximately 85 μm^2^, with a lateral length of about 14 μm and a depth of 2 μm. Indentation hardness and modulus were measured in all the samples (as-cast, homogenized, and aged) to assess the hardness changes due to heat treatment. Hardness and modulus values presented are derived from the CSM data and averaged between depths of 500 and 1900 nm.

### Microstructure characterization methods

SEM imaging, energy dispersive x-ray spectroscopy analysis using the Oxford Instrument XmaxN 150 detector, and electron backscatter diffraction analysis using the Oxford Instrument Nordlys Max detector were performed on a Zeiss Crossbeam 540 dual-beam instrument. Colocative EDX and EBSD maps were acquired simultaneously at 20 kV and 10 nA and a step size of 1 to 1.5 μm. 3D FIB sectioning was carried out on a Zeiss Crossbeam 540 dual-beam instrument using a using a 300-pA and 30-kV Ga + beam. A stack of 2D SEM images was acquired and visualized using the Thermo Fisher Scientific Avizo software. The detailed procedures have been described in (*50*).

TEM foils and APT needles were prepared using the in situ lift-out FIB method (*51*) on a Zeiss Crossbeam 540 FIB/SEM system. TEM foils were prepared using initial milling currents of 7000 to 300 pA at 30 kV and further thinned at 5 kV and 200 pA to a uniform thickness of ~50 nm, which is important for the correlative analysis using the EELS and TKD (*52*). HAADF images and EELS spectra were acquired on a Cs-corrected JEOL ARM 200F (cold field emission gun) operating at 200 kV equipped with a Quantum Gatan image filter. The convergence angle for EELS spectra acquisition was 31 mrad, and the collection angle was 41 mrad. APT specimens were analyzed in a CAMECA LEAP 5000XR with a laser energy of 0.5 nJ, a stage temperature of 50 K, and a repetition rate of 200 kHz. Data reconstruction was carried out using IVAS 3.6.8 software. On-axis TKD analysis was carried out on a Zeiss Merlin FEG-SEM system equipped with a Bruker e-flash high resolution EBSD detector and an OPTIMUS TKD head at an accelerating voltage of 30 kV, a beam current of 1.5 nA, and a step size of 5 and 10 nm.

### Theoretical analysis of precipitation hardening effects

As shown in Fig. 3, the intragranular precipitates in the TiVNbTa-4 sample have a volume fraction of around 3% and exhibit three distinct morphologies: plate-like, rod-like, and particle-like. Given the substantial difference between grain size and nanoindentation size, the hardening effect is calculated only from intragranular precipitates, excluding boundary precipitates. These intragranular precipitates are likely semicoherent and nonshearable, and the strengthening mechanism is thus based on Orowan looping mechanisms. The critical resolved shear stress (CRSS) needed for dislocations to bypass these obstacles is determined by considering the contributions due to all the nonshearable precipitates.

As a first approximation, we assume an equal volume fraction for these three types precipitates. The plate-like precipitate, measuring 1 μm in diameter and 50 nm in thickness, occupies 1% of the total volume fraction. The rod-like precipitate, with dimensions of 20 nm in diameter and 500 nm in length, also contributes 1% to the total volume fraction. Similarly, the particle-like precipitate, with a diameter of 60 nm, accounts for a volume fraction of 1%.

For a dispersion of spherical particles of uniform diameter *d*_particle_, distributed uniformly in the slip plane, the increase in the CRSS is determined as follows (*53*)

$$\text{Δ}\tau_{\mathrm{partile}}=\frac{Gb}{2\pi \sqrt{1-\nu }}\;\frac{1}{\lambda }\;\ln\left(\frac{d_{\mathrm{particle}}}{r_{c}}\right)$$

where *G* and ν represent the shear modulus and Poisson’s ratio of the matrix, respectively. They are related to the atomic fraction of all alloying elements (*54*), and the expressions are given as

$$G=\sum c_{i}G_{i},\;\nu =\sum c_{i}\nu_{i}$$

The values of *c_i_* for all elements are listed in Table 2, and the shear modulus and Poisson’s ratio for each element, denoted as *G_i_* and ν*_i_*, respectively, are provided in table S3. In addition, *d*_particle_ represents the equivalent diameter of the precipitate. *r*_c_ is the dislocation core radius, assumed to be 5**b**, where **b** stands for the magnitude of the Burgers vector. In the case of a BCC lattice, the Burgers vector is calculated as $\sqrt{3}/2a$, with *a* = 3.2318 Å represents the lattice parameter of the homogenized TiVNbTa sample (*14*). λ is the effective planar interobstacle spacing and can be estimated as follows (*54*)

$$\lambda =L_{p}-d_{p\mathrm{article}}=\left(\frac{0.779}{\sqrt{f_{\mathrm{particle}}}}-0.785\right)d_{\mathrm{particle}}$$

where *L*_p_ is the mean planar center-to-center interprecipitate spacing and *f*_particle_ is the volume fraction of the particle-like precipitate. Therefore, the CRSS increment resulting from the presence of particle-like precipitate is

$$\text{Δ}\tau_{\mathrm{partile}}=\frac{Gb}{2\pi \sqrt{1-\nu }}\frac{1}{\left(\frac{0.779}{\sqrt{f_{\mathrm{particle}}}}-0.785\right)d_{\mathrm{particle}}}\;\ln\left(\frac{d_{\mathrm{particle}}}{r_{c}}\right)$$

For plate-like precipitate, we adopt the strengthening model developed by Nie and Muddle (*55*)

$$\text{Δ}\tau_{\mathrm{plate}}=\frac{Gb}{2\pi \sqrt{1-\nu }}\;\frac{1}{0.931\sqrt{0.306\pi dt/f_{\mathrm{plate}}\pi d/}-1.061t}\;\ln\left(\frac{1.225t}{b}\right)$$

where *d* and *t* are the equivalent diameter and thickness of the plate-shaped precipitate, respectively, and *f*_plate_ is the corresponding volume fraction.

For rod-shaped precipitate, Zhu and Starke (*56*) proposed the following modification of the Orowan equation

$$\text{Δ}\tau_{\mathrm{rod}}=\frac{0.112Gb}{d_{\mathrm{rod}}}\;\ln\left(\frac{1.316d_{\mathrm{rod}}}{r_{c}}\right)(f_{\mathrm{rod}}^{1/2}+0.94f_{\mathrm{rod}}+2.44f_{\mathrm{rod}}^{3/2})$$

where *d*_rod_ is the equivalent diameter of the cross section of the rod-shaped precipitate and *f*_rod_ is the corresponding volume fraction.

Furthermore, when considering a mixture of n different types of precipitates, the increase in CRSS can be determined by (*57*)

$$\text{Δ}\tau_{p}={\left(\sum \text{Δ}{\tau_{i}}^{q}\right)}^{1/q}$$

where the value of the exponent *q* (with 1 ≤ *q* ≤ 2) depends on the relative strengthening effect of different types of precipitates. For similar strengthening effects among all precipitates, *q* = 2, leading to a phenomenological quadratic superposition approximation as follows

$$\text{Δ}\tau_{p}=\sqrt{\text{Δ}{\tau_{\mathrm{partile}}}^{2}+\text{Δ}{\tau_{\mathrm{plate}}}^{2}+\text{Δ}{\tau_{\mathrm{rod}}}^{2}}$$

Using this equation, the increase in CRSS can be derived as 19.25 MPa.

The resulted increase in Vickers hardness (*H*_V_ in kilograms per square millimeter) can be calculated from the increase in CRSS, based on the widely accepted relationship (*58*)

$$\text{Δ}\tau_{p}=\frac{T}{M}\text{Δ}H_{V}$$

where *M* represents the Taylor factor, for BCC materials discussed here, and a value of 2.73 is adopted. *T* is the Tabor factor with a value of 3.

To compare with the nanoindentation hardness, a relationship between the nanoindentation hardness *H*_IT_ and Vickers hardness *H*_V_, ∆*H*_IT_ = 1.25∆*H*_V_ is used (*59*). Therefore, the conversion from the increase in CRSS to the increase in nanoindentation hardness is

$$\text{Δ}H_{\mathrm{IT}}=\text{Δ}\tau_{p}\frac{1.25M}{T}$$

With this equation, the increase in nanoindentation hardness caused by the precipitates with a volume fraction of 3% is calculated as ∆*H*_IT_ = 217.48 MPa, representing 5.15% of the overall hardness of the TiVNbTa-4 sample. The calculations indicate that the rod-like precipitates contribute 17.42 MPa to the CRSS, while the plate-like and particle-like precipitates contribute 9.05 and 11.40 MPa, respectively. This suggests that rod-like precipitates play a dominant role in the precipitation hardening effect compared to the plate-like and particle-like precipitates.
