# Supplementary material for: Origin of age softening in the refractory high-entropy alloys
Source: Sci Adv. 2023 Dec 8;9(49):eadj1511. doi: 10.1126/sciadv.adj1511 (PMC10708198; doi:10.1126/sciadv.adj1511)
Supplement: Supplementary file 1 — Fig. S1 to S4 Table S1 to S3 [file sciadv.adj1511_sm.pdf]

Science Advances
AAAS

Supplementary Materials for

Origin of age softening in the refractory high-entropy alloys

Junliang Liu *et al.*

Corresponding author: Junliang Liu, jliu644@wisc.edu; David E. J. Armstrong, david.armstrong@materials.ox.ac.uk

Sci. Adv. **9**, eadj1511 (2023)
DOI: 10.1126/sciadv.adj1511

This PDF file includes:

Figs. S1 to S4
Tables S1 to S3

Table S1 APT chemical analysis. Composition of matrix in 1-day (TiVNbTa-2) and 40-day (TiVNbTa-4) aged samples

Element	TiVNbTa-2						TiVNbTa-4	
	#1	#2	#3	#4	#5	#6	#7	#8
Ti	26.00	25.80	25.90	26.10	25.90	26.10	25.20	25.73
V	25.60	25.50	25.70	25.70	25.50	25.90	25.88	26.73
Nb	24.90	24.90	24.70	25.20	24.90	24.80	25.57	26.02
Ta	22.10	22.70	22.30	21.80	22.60	21.80	22.55	20.49
O	0.81	0.78	0.72	0.71	0.77	0.73	0.30	0.42
N	0.41	0.35	0.48	0.34	0.34	0.47	0.22	0.17
C	0.08	0.06	0.06	0.07	0.06	0.08	0.04	0.05
Al	0.04	0.04	0.05	0.04	0.04	0.07	0.04	0.04
Ga	0.03	0.01	0.03	0.02	0.01	0.04	0.20	0.34
B	0.0009	0.0002	0	0	0	0.002	0	0
Sc	0	0	0	0	0	0	0.001	0.0004
Sum of C, N, O	1.30	1.19	1.26	1.12	1.17	1.28	0.56	0.64

Table S2 Calculated atomic size difference parameter (δ) for TiVNbTa alloys with different oxygen content.

	TiVNbTa	TiVNbTa-$O_{0.6}$	TiVNbTa-$O_{1.2}$
δ	3.93%	5.42%	6.58%

Table S3 Shear Modulus and Poisson's ratio for the elements using in calculations. Values are adapted from https://periodictable.com/

Elements	Shear Modulus, G (GPa)	Poisson’s ratio, ν
Ti	44	0.32
V	47	0.37
Nb	38	0.4
Ta	67	0.34

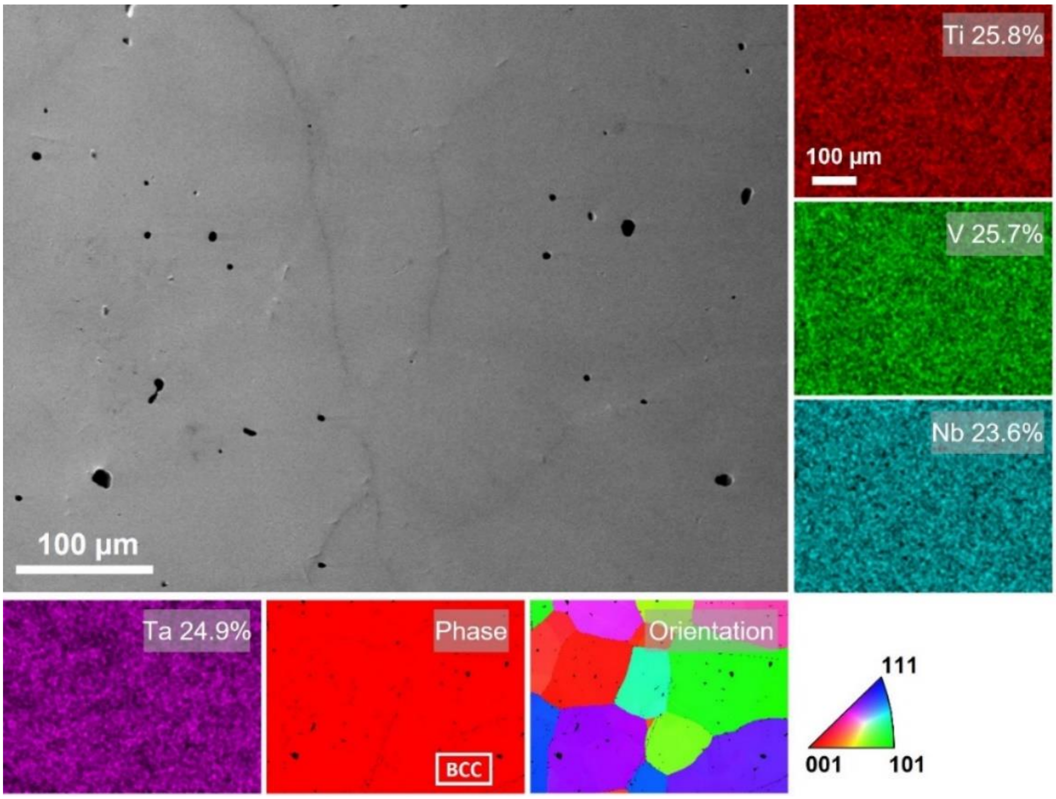

Fig. S1 Co-located SEM, EDX and EBSD maps from sample TiVNbTa-4. The unit for element concentrations labelled in the EDX maps is at. %.

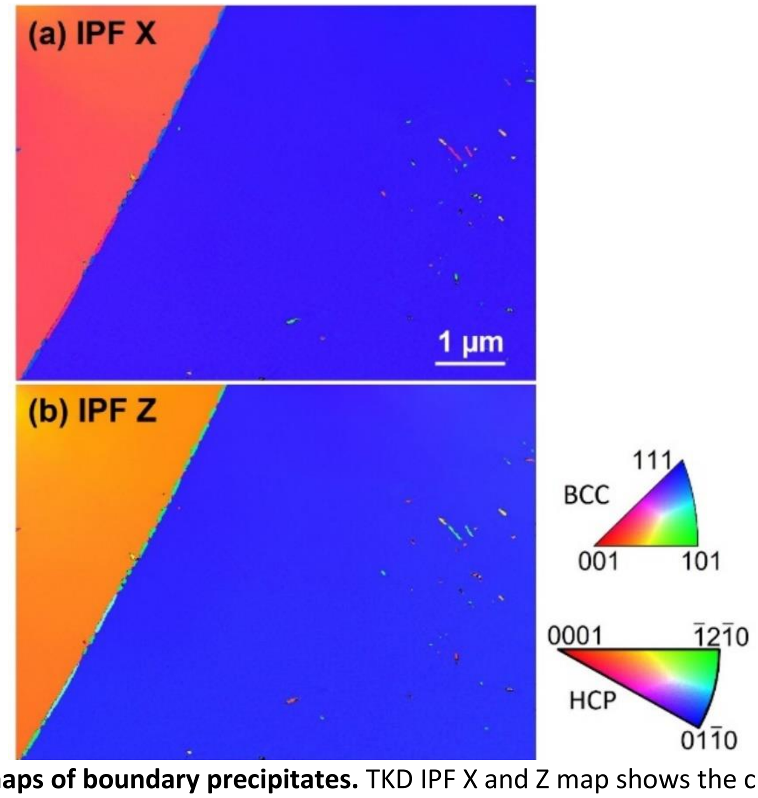

Fig. S2 TKD maps of boundary precipitates. TKD IPF X and Z map shows the crystallographic orientations for the precipitates and matrix in the sample TiVNbTa-4.

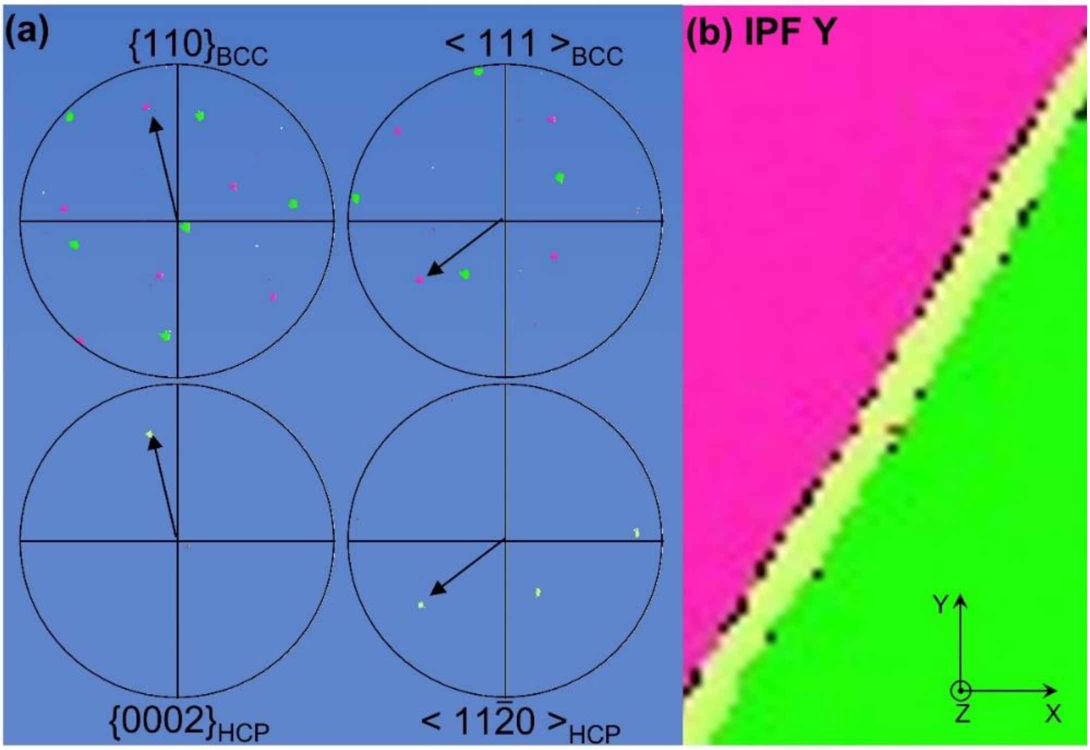

Fig. S3 TKD orientation relationship analysis in the region 2 in figure 5. (A) pole figures, **(B)** an enlarged orientation map cropped from figure 6 (d) showing the analysis region. The colour of points scattered to different angles in the pole figures in (a) is displayed in the same colour scheme in (b). The arrows in (a) indicated the aligned plane and direction for the two phases.

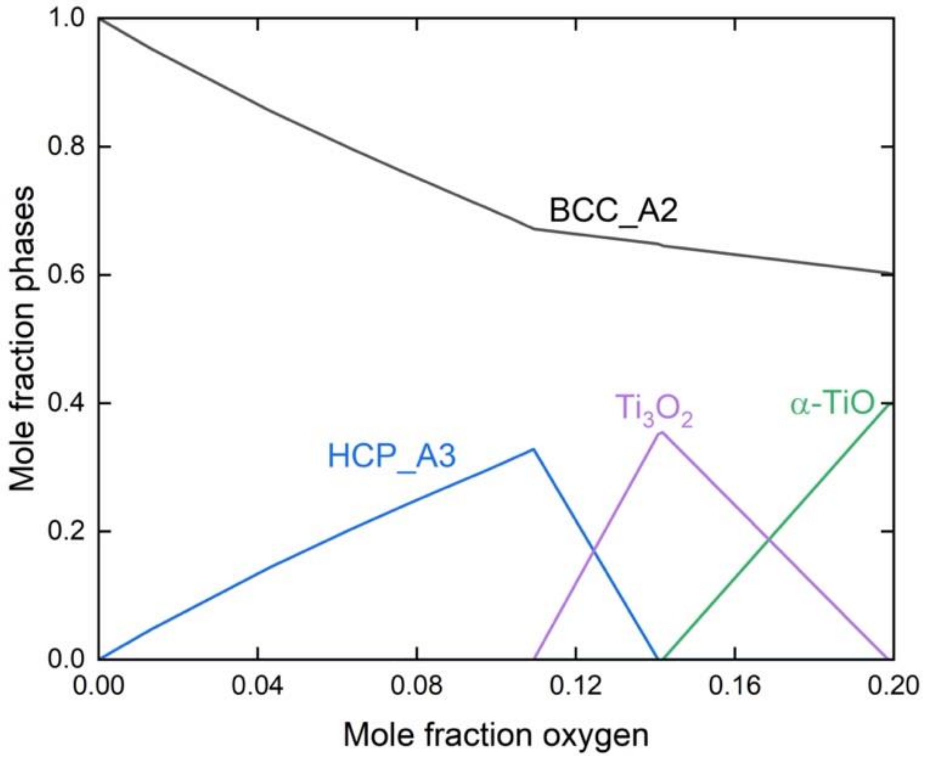

Fig. S4 Calculated equilibrium phase diagram. The phase fraction in the Ti-V-Nb-Ta system at 700°C as a function of oxygen content.
